# Complementary and alternative medicine use among pediatrics in Palestine: a cross-sectional study

**DOI:** 10.1186/s12887-021-02985-6

**Published:** 2021-11-11

**Authors:** Samah W. Al-Jabi, Mariam Khader, Islam Hamarsha, Dina Atallh, Sereen Bani-Odeh, Areen Daraghmeh, Shaima Bani-Mater, Sa’ed H. Zyoud

**Affiliations:** 1 Department of Clinical and Community Pharmacy, College of Medicine and Health Sciences, An-Najah National University, Nablus, 44839 Palestine; 2 Poison Control and Drug Information Center (PCDIC), College of Medicine and Health Sciences, An-Najah National University, Nablus, 44839 Palestine; 3 Clinical Research Centre, An-Najah National University Hospital, Nablus, 44839 Palestine

**Keywords:** Complementary and alternative medicine, Children, Pediatrics, Palestine

## Abstract

**Background:**

Recent use of complementary and alternative medicine (CAM) is growing in children worldwide, so there is a need to evaluate CAM’s use among pediatrics in Palestine. This study aimed to estimate the prevalence of CAM use among a sample of Palestinian children, investigate the factors that affect the use of CAMs, identify the types of CAM used, and assess the purposes of using them.

**Methods:**

A cross-sectional descriptive study of parents who had children aged 6 years and below was carried out. A convenient sample of about 420 participants was collected; from Primary care to Motherhood and Childhood Centers in Nablus city in Palestine. Parents who agreed to participate were asked to answer a survey that consists mainly of closed-ended questions. The analysis was performed using SPSS version 16.0.

**Results:**

The use of CAM was detected in all interviewers 420 (100%). The most common treatment used was herbal therapy (*n* = 400, 95.2%), and anise was the most common herbal therapy used (*n* = 334; 79.5%). A total of 371 (88.3%) of the respondents used CAM for digestive system problems. The main reason for using CAM was making the child more comfortable (*n* = 365; 86.9%). Parents who are 30 years or older were significantly using more CAM than younger parents (*P* = 0.001). In addition, regarding the number of children in the family, parents who have five children or more used more CAM subtypes significantly more than parents with less than this number (*P* = 0.025). Moreover, parents living in a refugee camp used more CAM than parents who lived in urban or rural areas (*P* = 0.031).

**Conclusions:**

Parents of children use CAM frequently. All parents used CAM, and physicians were not mainly among the sources of CAM information, and almost all parents were unaware of the side effects of CAM. Future research is necessary to direct pediatricians in formulating recommendations for children on CAM modalities, including possible risks and benefits and interactions with conventional medications.

## Background

Complementary and alternative medicine (CAM) is a formal method of health care in many areas of the ancient world and is expected to be integrated widely into the modern medical system and become part of it [1, 2]. CAM is a group of diverse medical and health care systems, practices, and products considered outside conventional medicine or used as an alternative to the traditional ones [3, 4]. However, it is not taught in traditional medical schools and is not applied in hospitals [5].

Many types of CAM are quite diverse in different areas of the world, and they are influenced by culture, history, use, level of education, and individual interests. Countries such as China, South Korea, and Vietnam have accepted the use of complementary medicine in their health systems [5]. CAM is commonly used among adults; also it has been reported to be used among children, especially among those with a parent who uses CAM for treatment or prevention of painful, chronic, recurrent, or incurable conditions [6]. People think that CAM use has many advantages over conventional medicine; they believe that CAM is more effective, safe, has a good patient/therapy relationship, and controls treatment. Moreover, the availability, acceptability, cost-effectiveness, and expectation of low side effects of CAM may increase the challenge of health care [7, 8].

The most common reason for using CAMs among pediatrics is parents’ fear of possible adverse effects of conventional medicine use. Thus, patients in several countries tend to use CAM despite being more efficacious prescribing therapy [6, 9, 10]. Furthermore, people think that conventional medicines have more side effects, and they do not prefer to go to doctors and wait for time to get treatment [7, 8]. It is known that the influence of parents, particularly mothers, on their children on how to take CAM and other medicines is highly effective [11, 12].

In pediatrics, the use of CAM is increasing worldwide. It has been used for different chronic illnesses such as asthma, arthritis, gastrointestinal diseases, neurological or developmental disorders, cancer, and other diseases. Despite the increased use of CAM among them, only a few parents tell physicians about CAM use; others don’t tell them, which may increase side effects and interactions with conventional medicines. Therefore, the attention of CAM use among pediatrics is increased to know its impact on their lives and develop evidence-based advice about their risks and benefits [13]. Due to the significant increase of CAM use among pediatrics worldwide, including Palestine, it is highly recommended to recognize and assess the prevalence of different CAM types among children in Palestine. This emphasizes the importance of understanding their worth and helps parents know its effect on their children. To date, the evaluation of CAM has received scant attention in the research literature in Palestine, considering aspects other than the use of CAM in children [8, 14–22]. To our knowledge, there are no available published data regarding the use of CAM in Palestinian children. Therefore, this study was performed to assess the use of different CAM types among children in Palestine, determine the main reported health problems treated with CAM among children, and evaluate parents’ main general views about CAM use. It is hoped that this research will contribute to a deeper understanding of this growing field of research by helping to prepare the strategies needed to improve the self-use of CAM. In addition, the findings can help develop educational programs to enhance parent counseling to avoid improper use of CAM and support university decision-makers to agree on the best courses to be given to medical students about CAM.

## Methods

### Study design and setting

A descriptive cross-sectional study was enrolled to determine the patterns of use, knowledge, and attitudes toward different CAM types among parents in Primary care for motherhood and childhood centers in Nablus city in Palestine. The data were collected in 2015.

### Population and sampling methods

This study is a descriptive cross-sectional study of parents who have children aged 6 years and below. Convenient sampling was used to select and recruit eligible parents. All parents aged 18 years and above whom the researchers met in the primary health care center in Nablus city were invited to participate in the study. Parents who agreed to participate were asked to answer a survey that consists mainly of closed-ended questions.

### Sample size

To calculate the sample size for this study, an automated software program “Raosoft sample size calculation” (http://www.raosoft.com/samplesize.html) was used. By assuming the response distribution to be 50% and allowing a 5% margin of error at a 95% confidence interval, the estimated sample size was 377 parents. To decrease erroneous results and increase the study reliability, the target sample size will be increased by 10%.

### Data collection and data collection form

A face-to-face interview was followed. Information collected included parents’ and children’s demographics, information about the types of CAM used in those children, the reasons for using CAM by parents, and information sources and knowledge of the possible side effects that may occur. Thus, the research team developed the study questionnaire through a comprehensive literature review and findings from previous studies to achieve our objectives [5, 23, 24]. In addition, the data collection form included questions based on the methodology of previously published studies and adapted to the Palestinian culture and customs [17, 18, 21, 22]. Our questionnaire consisted of questions related to demographic data including parent’s age, gender, level of education, income, marital status, number of children, youngest one age, number of children aged up to 6 years, locality, employment status, and health insurance. In addition, the questionnaire contained details relating to the use of CAM use in children regarding the following aspects: Parents were asked about the types of CAM they use with their children, how often they used CAM during the past 6 months, the health problems treated with CAM, how they got by know about the CAM used to their children, and reasons for CAM use. The parents were also asked if they told health care providers about the use of CAM and if they faced any complications or side effects from CAM use. Finally, the parents were asked about general beliefs of CAM.

The questionnaire was pretested among 30 parents to ensure the transparency and accuracy of the questions. The expert team (three clinical pharmacists) assessed the accuracy and interpretation of the pretest group’s completed questionnaires and the final draft was generated after removing, adding, and changing certain questions.

### Ethical approval

The Institutional Review Boards (IRB) of An-Najah National University and the Ministry of Health (MOH) approved all aspects of the research procedure, including access to and use of clinical information for patients, prior to the initiation of this research. In addition, only participants who agreed to participate in the study (a verbal consent form) after they were explained the nature and objectives of the study were included. The data collection form has been distributed by researchers to the parents after obtaining written permission from the MOH). To increase the response rate, all participants were asked face-to-face as they were personally briefed by the researchers about the study and its significance. Moreover, we confirmed that the collected data will be used for clinical research only, while the information will be confidential and will not be used for any purpose other than the study.

### Statistical analysis

The data was entered and analyzed using version 16 of the Statistical Package for Social Sciences (SPSS) software. For continuous variables, data were expressed as means ± SD or medians (interquartile range), and as frequencies (percentage) for categorical variables. The numerical variables were tested for normality using the Kolmogorov-Smirnov test. The number of CAM used was compared with socio-demographic parameters. Kruskal-Wallis and Mann-Whitney U tests were used to test the statistical significance of differences between the groups. In which the Mann-Whitney U test was used to compare the means of the continuous variables of two categories. In contrast, the Kruskal-Wallis H test was used to compare between means of continuous variables for more than two categories. All results were regarded as statistically significant at *P* < 0.05.

## Results

### Socio-demographic characteristics

A total number of 420 childhood parents participated in this study**.** In this study, 432 parents were interviewed. However, 12 parents did not respond. Thus, the response rate of the current study was 97%. The sample was collected from three centers; from each, 140 (33.33%) parents were interviewed. Table 1 shows the demographic characteristics of the study participants. The mean age (± SD) of all respondents was 28.8 ± 6.4 years, ranging from (19-52) years. Furthermore, the majority of them were female (*n* = 414, 98.6%) and reported that they were married (*n* = 416, 99%)**.** In addition, the majority of them (*n* = 168, 40.0%) were completed secondary education, followed by 153 (36.4%) who completed their university education. Moreover, 346 (82.4%) of parents were unemployed. And regarding the living place, the majority of parents were living in urban areas (*n* = 285, 67.9%).

**Table 1:** Sociodemographic characteristics of the study participants

Variable	Frequency (%) ***N*** = 420
**Age (year)**	28.8 ± 6.4
**Gender**	
Male	6 (1.4)
Female	414 (98.6)
**Level of education**	
Illiterate	7 (1.7)
Primary	86 (20.5)
Secondary	168 (40.0)
University	153 (36.4)
Postgraduate	6 (1.4)
**Income**	
Less than 2000 NIS^a^)	80 (19.0)
Moderate (2000-5000 NIS)	301 (71.7)
High (More than 5000 NIS)	39 (9.3)
**Marital status**	
Married	416 (99)
Divorced	2 (0.5)
Widowed	2 (0.5)
**Number of children**	3 (2-4)
**Youngest child age (year)**	1 (0.44-2)
**Number of children up to 6 years**	2 (1-2)
**Locality**	
Urban	285 (67.9)
Rural	105 (25)
Camp	30 (7.1)
**Employment status**	
Unemployed	346 (82.4)
Employed	62 (14.8)
Employed in health service	7 (1.7)
Previously employed	5 (1.2)
**Health insurance**	
Yes	327 (77.9)
No	93 (22.1)

^a^1 USD to NIS = 3.44496 New Israeli Shekels

Regarding the patient’s family income, 301 (71.7%) parents interviewed had moderate family income (2000-5000 NIS). And the majority of them have health insurance 327 (77.9%). Regarding the number of children their family had, the median (interquartile range) of the number of children in each family was 3 (2 – 4) children, ranging from (1 - 9) children. The median (interquartile range) of the number of children aged up to 6 years is 2 (1 – 2), ranging from (1-6) years. In addition, the median (interquartile range) of the youngest one is 1 (0.4 – 2) years, ranging from (0.04 - 6) years.

### CAM use among children

In our survey, the use of CAM was detected in all interviewers (*n* = 420, 100%). Different types of CAM were detected among them. Table 2 summarizes the types of CAM that were used among their children. According to Table 2, results presented that herbal therapy is the most commonly used (*n* = 400, 95.2%), followed by Quran reading (*n* = 381, 90.7%), Oil rub (*n* = 364, 86.6%), Prayer (*n* = 351, 83.6%), and Hot/Cold application (*n* = 347, 82.6%).

**Table 2:** Types of complementary and alternative medicine used among children

Types of complementary and alternative medicine used	Frequency (%) ***N*** = 420
Herbal therapy	400 (95.2)
Quran reading	381 (90.7)
Oil rub	364 (86.7)
Prayer	351 (83.6)
Hot/Cold applications	347(82.6)
Vitamin and minerals	232 (55.2)
Massage	195 (46.4)
Zamzam water	177 (42.1)
Honey	145 (34.5)
Animal sources	132 (31.4)
Music	129 (30.7)
Aromatherapy	84 (20)
Popular Recipes	65 (15.5)
Blood letting	7 (1.7)
Cupping	6 (1.4)
Acupuncture	5 (1.2)
Cauterization	2 (0.5)

### Types of herbal use among children

When the parents were asked about the types of herbal use among their children, they documented that the most commonly used herb is Anise (*Pimpinella anisum*) in 334 (79.5%), followed by Sage (*Salvia officinalis*) in 261 (62.1%) and Chamomile (*Anthemis nobilis*) in 198 (47.1%); (Table 3).

**Table 3:** Types of herbal use among children

Types of herbals	Frequency (%) ***N*** = 420
Anise (*Pimpinella anisum*)	334 (79.5)
Sage (*Salvia officinalis*)	261 (62.1)
Chamomile (*Anthemis nobilis*)	198 (47.1)
Peppermint (*Mentha piperita*)	137 (32.6)
Mixed herbals	103 (24.5)
Thyme (*Thymus serpyllum*, *Thymus vulgaris*)	102 (24.3)
Cumin (*Cuminum cyminum*)	74 (17.6)
Potato (*Solanum tuberosum*)	67 (16)
Starch	58 (13.8)
Ginger (*Zingiber officinale*)	46 (11)
Fenugreek (*Trigonella foenum-graecum*)	45 (10.7)
Blooms water	33 (7.9)
Fennel (*Foeniculum vulgare*)	32 (7.6)
Rosemary (*Rosmarinus officinalis*)	31 (7.4)
Guava (*Psidium guajava* L.) leaves	26 (6.2)
Artemisia (*Artemisia absinthium)*	23 (5.5)

### Health problems treated with CAM

More than half of the parents (*n* = 238, 56.7%) confirmed that they sometimes used CAM in their children for various health problems. Table 4 shows the common health problems that parents chose CAM to treat. A total of 371 (88.3%) of the respondents used CAM for digestive system problems. In addition, 324 (77.1%) parents used CAM for respiratory system problems. Furthermore, sleep problems and skin problems (33.1 and 23.1% respectively) were the third and fourth most common health problems for which parents used CAM in their children.

**Table 4:** Health problems treated with complementary and alternative medicine

Reported health problem treated with complementary and alternative medicine	Frequency (%) ***N*** = 420
Digestive system problem	371 (88.3)
Respiratory system problem	324 (77.1)
Sleep problem	139 (33.1)
Skin problem	97 (23.1)
Musculoskeletal system problem	32 (7.6)
Urinary system problem	20 (4.8)
Nervous system problem	17 (4)

### Sources of knowledge about CAM use

Parents were asked about the sources from which they got knowledge for using CAM in their children. The results show that relatives and friends were the most common source for parents’ knowledge about CAM use (290, 69%). In addition, 175 (41.7%) took knowledge about CAM use from their children’s doctors (Table 5).

**Table 5:** Sources of knowledge about complementary and alternative medicine use

Sources of knowledge about complementary and alternative medicine use	Frequency (%) ***N*** = 420
From a relative/friend	290 (69)
From the child’s doctor	175 (41.7)
From some other person	172 (41)
From media	171 (40.7)
From a pharmacist or nurse or any other doctor	101 (24)
Found out about it by my experience	78 (18.6)
From CAM store salesperson	16 (3.8)

### Reasons for CAM use

Table 6 represents the reasons for CAM use among children. Making the child more comfortable is the leading cause of using CAM (*n* = 365, 86.9%), followed by promoting health and preventing disease (*n* = 184, 43.8%).

**Table 6:** Reasons for complementary and alternative medicine use

Stated reasons for complementary and alternative medicine use	Frequency (%) ***N*** = 420
Making child comfortable	365 (86.9)
Promoting health and preventing disease	184 (43.8)
Supporting medical treatment	137 (32.6)
Reducing the side effects of medical treatment	65 (15.5)
No satisfaction with medical treatment	57 (13.6)

### Awareness of health care providers about CAM use

Parents were asked about the awareness of health care providers, including physicians and nurses, about CAM use in children. Nearly half of the parents told their health care providers about using CAM in their children (*n* = 212, 50.5%).

### Usefulness of CAM

The current study results found that 385 (91.5%) of parents found that CAM use in their children was useful.

### Complications of CAM use

Parents were asked if they thought that CAM might cause any complications or side effects; 369 (87.9%) believed that CAM had no complications or side effects.

### Associations between socio-demographic characteristics and the use of CAM

The median (interquartile range) of the CAM subtypes used by children was 7 (6 – 9) subtypes, with a range of 2 – 12 subtypes. Parents who are 30 years or older were significantly using more CAM than younger parents (8 (6 – 9) versus 7 (5 – 9), *P* = 0.001). In addition, regarding the number of children in the family, parents who have five children or more users use more CAM subtypes 8 (7 – 9) significantly more than parents with less than this number (*p* = 0.025). Moreover, parents living in camp 8 (7 – 10) used more CAM than parents who lived in urban 7 (5 – 9) or rural 7 (6 - 9) areas (*p* = 0.031). The study found that parents having health insurance used significantly more CAM (*P* = 0.005).

On the other hand, no significant association between CAM use and parents’ level of education, income, marital status, and employment status (*P* > 0.05).

### General views of parents CAM

Table 7 clarifies parents’ general view on CAM among the study participants; 293 (69.7%) of them disagreed / strongly disagreed that CAM may be used just in adults, not in children. In addition, 242 (57.6%) parents agreed / strongly agreed that some health problems in children could be better controlled by CAM than conventional medications recommended by a physician. Furthermore, 217 (51.7%) of parents disagreed/ strongly disagreed that CAM should not be used in combination with conventional medications recommended by physicians. Most parents (*n* = 300, 71.4%) disagreed/ strongly disagreed that CAM could cause side effects in children. Furthermore, 311 (64%) of parents agreed/ strongly agreed that CAM is usually safer in children than conventional medications recommended by physicians.

**Table 7:** General views of parents about complementary and alternative medicine

I believe/think	Strongly Disagree	Disagree	Uncertain/Don’t know	Agree	Strongly Agree
CAM may be used just in adults, not in children.	40 (9.5)	253 (60.2)	28 (6.7)	70 (16.7)	29 (6.9)
Some health problems in children could be better controlled by CAM than conventional medications recommended by a physician	30 (7.1)	109 (26)	39 (9.3)	205 (48.8)	37 (8.8)
CAM should not be used in combination with conventional medications recommended by physicians	29 (6.9)	188 (44.8)	37 (8.8)	138 (32.9)	28 (6.7)
CAM could cause side effects in children	35 (8.3)	300 (71.4)	16 (3.8)	61 (14.5)	8 (1.9)
CAM is usually safer in children than conventional medications recommended by physicians	12 (2.9)	61 (14.5)	36 (8.6)	234 (55.7)	77 (18.3)
CAM users should notify their physicians that they used CAM	12 (2.9)	88 (21)	10 (2.4)	249 (59.3)	61 (14.5)
CAM is usually more effective in children than medications recommended by physicians	20 (4.8)	175 (41.7)	70 (16.7)	130 (31)	25 (6)
It is an essential for more information on the numerous CAM therapies available.	2 (0.5)	21 (5)	1 (0.2)	224 (53.3)	172 (41)

Furthermore, 249 (74%) of parents agreed/ strongly agreed that CAM users should notify their physicians that they used CAM, 195 (46.5%) of parents disagreed / strongly disagreed that CAM is usually more effective in children than medications recommended by physicians. Moreover, 396 (94.3%) of parents agreed / strongly agreed that it is essential for more information on the numerous CAM therapies accessible (Table 7).

## Discussion

Many researchers worldwide have studied CAM use among pediatric patients, and this is the first study that focused on CAM use in pediatrics aged 6 years and below in Palestine. All parents reported the use of CAM in their children, and this result is because the current study does not focus on certain diseases, and most CAM subtypes were included in addition to the effect of culture.

The prevalence of CAM used in previous studies from Western countries conducted in Italy [25], Finland [10], and Canada [26], were 18-38, 11%, and 59-74%, respectively. In the Italian study, the most common CAM methods used were homeopathy, acupuncture, phytotherapy, traditional Chinese medicine, chiropractic, osteopathy, and anthroposophical were considered [25]. Furthermore, at a university hospital in Mexico, 45% of all parents mentioned that they used CAM with their children who suffer from hematologic problems [27].

At the *Neurology Clinic* at *King Abdullah University Hospital*, about 56% of parents who came with children with neurologic diseases such as epilepsy, cerebral palsy, and congenital brain malformations used CAM with their children [28].

The rate of CAM use in Palestine seems to be higher than that in other countries because parents often think that CAM use is more natural, safer, and less invasive than conventional medicine. Moreover, as mentioned before, our survey was comprehensive in two aspects, the first is according the reasons for CAM use which are not specific and include general health problems, and the second is that parents were asked about CAM which includes a wide range of CAM subtypes whether it is a practice like a massage or a product like a herb.

In the current study, herbal therapy is the most commonly used CAM among parents (400, 95.2%), followed by Quran reading (381, 90.7%), oil rub (364, 86.6%), and prayer (351, 83.6%). These results were consistent with the results obtained from a study conducted by Sawalha [8] among adults from the northern part of Palestine, which found that herbal therapy and prayers were the most CAM types. In addition, a study in Turkey that explored the use of CAM among Turkish children found that herbal therapy was the most common CAM used [23].

A study in Germany that was developed to assess CAM use among healthy children and children with chronic medical conditions found that homeotherapy was the most therapy used [13]. In the United Kingdom, a study found that massage, aromatherapy, and homeotherapy were the main CAM types used for healthy children and among children with chronic illness [29].

It is expected that herbs are among the most commonly used CAM because many people believe that herbs are natural and always safe and suitable for their children, but this is not necessarily true. Moreover, these herbs are almost cheap and available. In addition, prior to recorded history, plants were used to treat illness; ancient Chinese and Egyptian papyrus writings identify medicinal uses for plants as early as 3000 BC [30], so their use moves from generation to another until it reaches us.

According to reading al Quran and prayers, as most or even all study parents are Muslims, they believe that Quran is a miracle and it is God’s word that has a rule in healing, and many verses in Quran mention that “*And We send down of the Quran that which is a healing and a mercy to those who believe” (Quran, Surah Al-Israa, 17:82).*

Regarding the health problems that were treated by CAM, in the current study, digestive and respiratory health problems were the most commonly treated conditions by CAM. In addition, 86.9% of parents reported that they used CAM to make their children more comfortable. These results are consistent with a study in Saudi Arabia conducted by Ashraf et al. [31]. They found that CAM is used more often in children with gastrointestinal and respiratory tract symptoms. In the Netherlands, CAM was used mainly to treat children with headaches, chronic fatigue, and parents’ desire to make their children feel better [32]. Furthermore, in Turkey, CAM was used for children with respiratory and digestive system problems with percentages of 49 and 25%, respectively, and 59% of parents used CAM mainly to make their children more comfortable and 25% to support prescribed drugs [23].

Regarding the source of information from which the parents received information about CAM, about 96% of parents mentioned that relatives and friends were the main sources of information about CAM use. This is consistent with a previous study by (Ozturk and Karayagiz, [23] which concluded that family and friends were the primary sources of information for CAM use in pediatrics in Turkey.

In Iran, in a study that evaluated the prevalence of use of CAM in children, relatives (72%) and neighbors (50%) were the most sources of knowledge about CAM for parents [5]. However, this is with study in Italy, where physicians were the source of information about CAM and were prescribed by them [33].

Most interviewers in our study (91.5%) found that CAM was useful and 87.9% of them believed that CAM has no complications or side effects. These attitudes were similar to a study in Iran, in which only 1.3% of mothers identified that CAM might have some side effects or complications [5]. In addition, in a study to assess CAM’s use among patients attending neurology clinics in Canada, most participants thought that CAM was beneficial, with little to no harm involved [26].

The safety or effectiveness of CAM in children has not been tested, and side effects may differ from those seen in adults. Children’s metabolism and their immune, digestive, and central nervous systems are still evolving and maturing, so parents should be aware of this when using CAM with their children. In a study by Lim and colleagues [34] aimed to determine the types of adverse events associated with CAM use during the period between (2001-2003) as seen by Australian pediatricians, 46 reports of adverse events were recorded to be caused by CAM use. In a Canadian study, 7% of pediatricians and pediatric subspecialists reported seeing adverse events and 18% reported cases of delayed diagnosis or treatment due to using CAM [4].

In this study, the statistical analysis showed that parents’ age was a significant factor affecting the number of CAM used; parents who are 30 years or older use CAM in their children more than other ages. This result is consistent with a study in Iran by Fesharakinia and Abedini, [5] where parents between the age of 30-40 years use CAM in their children more than other age categories. Moreover, parents who have five children or more used more CAM subtypes than parents who have fewer children. These results may be explained as those parents have more experience and gather more knowledge and awareness during their life regarding child diseases and CAM use, and they are still practicing what was done in the past as people before depend more on CAM therapy. Additionally, parents who have many children found it more cost-effective to use CAM than visiting doctors.

In addition, our study found that parents living in the refugee camp used more CAM subtypes than parents living in urban or rural areas; this result is consistent with a previous study conducted by Ali-Shtayeh et al., [14] that found the use of CAM differed significantly between residents of refugee camps versus residents of urban or rural areas where camps residents used more CAM than others. This can be explained that families living in camps may be poorer. Thus, they depend mainly on CAM rather than on medications.

In addition to that, CAM use was higher in those with health insurance, like a study conducted in a tertiary children’s hospitals in Australia where CAM use was higher in those with health insurance [34].

Furthermore, half of the parents said they make physicians or nurses aware of their CAM use. Such a study in Canada explored the use of CAM among children attending neurology clinics, where 57% discussed using CAM with their physician [26]. While in Australia and Newzealand, 63 and 77% of parents reported that CAM use had not been discussed with their treating doctor [34, 35].

It is necessary to tell healthcare professionals about CAM use in the pediatric patient, and physicians should be attentive when prescribing medication and ask parents if they use CAM. This is important because concurrent use of CAM and conventional medications are widespread and carry a potential risk to patients who may be susceptible to drug interactions.

### Strength and limitations

This is the first study that was conducted in Palestine that focused on assessing CAM use among pediatrics in Palestine. Face-to-face interview methodology was used in the study; it gives more accurate screening, can capture verbal and nonverbal answers, keep the patient focused while answering, and capture emotions and behavior.

Nevertheless, there were some limitations to the study. These limitations were associated with the poor recall of CAM experience and use. It is unfortunately that our study could not identify the outcomes of CAM use in this group of population, such as undesirable or side effects of CAM use. In addition, direct parental interviewing by researchers might involve a bias for parents who may have wished to respond privately. However, the questions of the study can be answered without any embarrassment.

## Conclusions

Complementary and alternative medicine is used commonly in pediatric patients in Palestine. Herbal therapy, especially “Anise” was the most common CAM used. Regarding the diseases treated with CAM, digestive system problems were the most common problems, and making the child more comfortable is the most common reason for CAM use. On the other hand, most parents felt that CAM use was helpful, with few or no associated adverse effects, so it is highly recommended to aware people about the proper use of CAM. The most common source of information for CAM among the study parents was information from relatives and friends, and nearly half of the parents did not tell the physician about CAM use. The results of this research have a variety of substantial consequences for future practice, including 1) complementary and alternative medicine use in the community must be improved through better parent and physician education of appropriate CAM use; 2) It is recommended for physicians to be aware of patients using CAM and ask about CAM use while taking a patient history, and 3) Pharmacists and clinical pharmacists have a law of consciousness to raise the risk of encounters with conventional medicines.

## Data Availability

The datasets used and/or analyzed during the current study are available from the corresponding author on reasonable request.

## Abbreviations

CAM: Complementary and Alternative Medicine
IRB: Institutional Review Board
MOH: Ministry of Health.

## References

[1] Azaizeh H, Saad B, Cooper E, Said O (2010). Traditional Arabic and Islamic medicine, a re-emerging health aid. Evid Based Complement Alternat Med.

[2] National Center for Complementary and Integrative Health (2015). Complementary, alternative, or integrative health: What’s in a name?.

[3] Tabish SA (2008). Complementary and alternative healthcare: is it evidence-based?. Int J Health Sci (Qassim).

[4] Vohra S, Brulotte J, Le C, Charrois T, Laeeque H (2009). Adverse events associated with paediatric use of complementary and alternative medicine: results of a Canadian Paediatric Surveillance Program survey. Paediatr Child Health.

[5] Fesharakinia A, Abedini M (2014). Prevalence of using complementary and alternative medicine in children and its related factors in East Iran. Iran J Pediatr.

[6] Simpson N, Roman K (2001). Complementary medicine use in children: extent and reasons. A population-based study. Br J Gen Pract.

[7] Ernst E (2000). The role of complementary and alternative medicine. BMJ.

[8] Sawalha AF (2007). Complementary and alternative medicine (CAM) in Palestine: use and safety implications. J Altern Complement Med.

[9] Menniti-Ippolito F, Gargiulo L, Bologna E, Forcella E, Raschetti R (2002). Use of unconventional medicine in Italy: a nation-wide survey. Eur J Clin Pharmacol.

[10] Siponen SM, Ahonen RS, Kettis A, Hameen-Anttila KP (2012). Complementary or alternative? Patterns of complementary and alternative medicine (CAM) use among Finnish children. Eur J Clin Pharmacol.

[11] Bush PJ, Iannotti RJ (1990). A children’s health belief model. Med Care.

[12] Hameen-Anttila KP, Niskala UR, Siponen SM, Ahonen RS (2011). The use of complementary and alternative medicine products in preceding two days among Finnish parents - a population survey. BMC Complement Altern Med.

[13] Gottschling S, Gronwald B, Schmitt S, Schmitt C, Langler A, Leidig E, Meyer S, Baan A, Shamdeen MG, Berrang J (2013). Use of complementary and alternative medicine in healthy children and children with chronic medical conditions in Germany. Complement Ther Med.

[14] Ali-Shtayeh MS, Jamous RM, Jamous RM (2012). Complementary and alternative medicine use amongst Palestinian diabetic patients. Complement Ther Clin Pract.

[15] Ali-Shtayeh MS, Jamous RM, Jamous RM, Salameh NM (2013). Complementary and alternative medicine (CAM) use among hypertensive patients in Palestine. Complement Ther Clin Pract.

[16] Ali-Shtayeh MS, Jamous RM, Salameh NM, Jamous RM, Hamadeh AM (2016). Complementary and alternative medicine use among cancer patients in Palestine with special reference to safety-related concerns. J Ethnopharmacol.

[17] Salah AO, Salameh AD, Bitar MA, Zyoud SH, Alkaiyat AS, Al-Jabi SW (2020). Complementary and alternative medicine use in coronary heart disease patients: a cross-sectional study from Palestine. BMC Complement Med Ther.

[18] Samara AM, Barabra ER, Quzaih HN, Zyoud SH (2019). Use and acceptance of complementary and alternative medicine among medical students: a cross sectional study from Palestine. BMC Complement Altern Med.

[19] Shawahna R (2018). Combining and using the Utrecht method and the analytic hierarchy process to facilitate professional and ethical deliberation and decision making in complementary and alternative medicine: a case study among a panel of stakeholders. Evid Based Complement Alternat Med.

[20] Shawahna R, Al-Atrash M (2019). What do primary healthcare providers and complementary and alternative medicine practitioners in Palestine need to know about exercise for cancer patients and survivors: a consensual study using the Delphi technique. Evid Based Complement Alternat Med.

[21] Shraim NY, Shawahna R, Sorady MA, Aiesh BM, Alashqar GS, Jitan RI, Abu Hanieh WM, Hotari YB, Sweileh WM, Zyoud SH (2017). Community pharmacists’ knowledge, practices and beliefs about complementary and alternative medicine in Palestine: a cross-sectional study. BMC Complement Altern Med.

[22] Zyoud SH, Al-Jabi SW, Sweileh WM, Tabeeb GH, Ayaseh NA, Sawafta MN, Khdeir RL, Mezyed DO, Daraghmeh DN, Awang R (2016). Use of complementary and alternative medicines in haemodialysis patients: a cross-sectional study from Palestine. BMC Complement Altern Med.

[23] Ozturk C, Karayagiz G (2008). Exploration of the use of complementary and alternative medicine among Turkish children. J Clin Nurs.

[24] Robert Gordon University (2015). Complementary & Alternative Medicine (CAM) use in children in North-East Scotland: a parent survey.

[25] Dolceamore TR, Altomare F, Zurlo F, Miniero R (2012). Use of alternative-complementary-medicine (CAM) in Calabrian children. Ital J Pediatr.

[26] Galicia-Connolly E, Adams D, Bateman J, Dagenais S, Clifford T, Baydala L, King WJ, Vohra S (2014). CAM use in pediatric neurology: an exploration of concurrent use with conventional medicine. PLoS One.

[27] Jaime-Perez JC, Chapa-Rodriguez A, Rodriguez-Martinez M, Colunga-Pedraza PR, Marfil-Rivera LJ, Gomez-Almaguer D (2012). Use of complementary and alternative medicine by patients with hematological diseases experience at a university hospital in Northeast Mexico. Rev Bras Hematol Hemoter.

[28] Aburahma SK, Khader YS, Alzoubi K, Sawalha N (2010). Complementary and alternative medicine use in a pediatric neurology clinic. Complement Ther Clin Pract.

[29] McCann LJ, Newell SJ (2006). Survey of paediatric complementary and alternative medicine use in health and chronic illness. Arch Dis Child.

[30] Seethalakshmi TS, Kandhimathinathan S, Rajan K, Manimala E (2014). Herbal medicine research–a scientometric view. Eur Acad Res.

[31] Ashraf S, Rahman AJ, Satwani H, Naz F, Abbas K, Hassan A (2010). Trend of complementary therapies in paediatric age group. J Pak Med Assoc.

[32] Vlieger AM, van de Putte EM, Hoeksma H (2006). The use of complementary and alternative medicine in children at a general paediatric clinic and parental reasons for use. Ned Tijdschr Geneeskd.

[33] Filippo M, Oliverio AC, Altomare F, Zurlo F, Savino F, Oggero R, Dolceamore TR, Miniero R (2013). Review on the use of complementary medicine in pediatrics: an interregional study. Minerva Pediatr.

[34] Lim A, Cranswick N, Skull S, South M (2005). Survey of complementary and alternative medicine use at a tertiary children's hospital. J Paediatr Child Health.

[35] Wilson K, Dowson C, Mangin D (2007). Prevalence of complementary and alternative medicine use in Christchurch, New Zealand: children attending general practice versus paediatric outpatients. N Z Med J.

